# Thromboprophylaxis in primary shoulder arthroplasty does not seem to prevent death: a report from the Norwegian Arthroplasty Register 2005–2018

**DOI:** 10.1080/17453674.2021.1906595

**Published:** 2021-04-06

**Authors:** Randi M Hole, Anne Marie Fenstad, Jan-Erik Gjertsen, Stein A Lie, Ove Furnes

**Affiliations:** a Norwegian Arthroplasty Register, Department of Orthopedic Surgery, Haukeland University Hospital , Bergen ;; b Department of Clinical Medicine, University of Bergen ;; c Department of Clinical Dentistry, University of Bergen , Norway

## Abstract

Background and purpose — There is still no consensus on whether to use thromboprophylaxis as a standard treatment in shoulder replacement surgery. We investigated the use of thromboprophylaxis reported to the Norwegian Arthroplasty Register (NAR). The primary endpoint was early mortality after primary shoulder arthroplasty with and without thromboprophylaxis. Secondary endpoints included revisions within 1 year and intraoperative complications.

Patients and methods — This observational study included 6,123 primary shoulder arthroplasties in 5,624 patients reported to the NAR from 2005 to 2018. Cox regression analyses including robust variance analysis were performed with adjustments for age, sex, ASA score, diagnosis, type of implant, fixation, duration of surgery, and year of primary surgery. An instrumental variable Cox regression was performed to estimate the causal effect of thromboprophylaxis.

Results — Thromboprophylaxis was used in 4,089 out of 6,123 shoulder arthroplasties. 90-day mortality was similar between the thromboprophylaxis and no thromboprophylaxis groups (hazard ratio (HR) = 1.1, 95% CI 0.6–2.4). High age (> 75), high ASA class (≥ 3), and fracture diagnosis increased postoperative mortality. No statistically significant difference in the risk of revision within 1 year could be found (HR = 0.6, CI 0.3–1.2). The proportion of intraoperative bleeding was similar in the 2 groups (0.2%, 0.3%).

Interpretation — We had no information on cause of death and relation to thromboembolic events. However, no association of reduced mortality with use of thromboprophylaxis was found. Based on our findings routine use of thromboprophylaxis in shoulder arthroplasty can be questioned.

Shoulder arthroplasty (SA) has gained wide acceptance as treatment for a variety of shoulder conditions, and the annual incidence rates are increasing (Lubbeke et al. 2017). Venous thromboembolism (VTE) is a recognized complication after hip and knee arthroplasties (Lie et al. 2002) but has been considered rare after SA. The number of reports of VTE after SA has increased with increasing number of SAs performed (Lyman et al. 2006, Jameson et al. 2011) and fatal outcome has also been reported (Saleem and Markel 2001, Madhusudhan et al. 2009). The true risk of VTE after SA has not been determined, and even though some studies suggest that the risk equals that of lower limb arthroplasty (Willis et al. 2009), most studies find a lower risk in the upper extremities (Isma et al. 2010, Saleh et al. 2013). Chemical thromboprophylaxis reduces the rates of symptomatic VTE following lower limb arthroplasty and is supposed to reduce mortality from thromboembolic complications (Dahl 1998, Senay et al. 2018). Thromboprophylaxis remains controversial among surgeons because it may carry a higher risk of bleeding, wound complication, and reoperation after orthopedic surgery (Kwong et al. 2012).

Guidelines on thromboprophylaxis exist in Norway and in other countries (SIGN 2010, Falck-Ytter et al. 2012, Kristiansen et al. 2014, National Institute for Health and Clinical Excellence 2018, Samama et al. 2018). While thromboprophylaxis is recommended for all patients undergoing hip or knee arthroplasties, there are still no evidence-based guidelines specific for SA. Due to the low number of SAs performed and the low rate of deaths due to thromboembolic events, a randomized trial would not be feasible. Hence, the best option to study the effect of thromboprophylaxis is large cohort studies (Fender et al. 1997). Using an observational population-based design with data from the Norwegian Arthroplasty Register (NAR) we studied the use of thromboprophylaxis in patients undergoing SA. Our primary endpoint was the influence of thromboprophylaxis on 90-day mortality. Secondary endpoints were intraoperative bleeding complications and revision due to all causes and due to infection within 1 year.  

## Patients and methods

This study was performed according to the Reporting of studies Conducted using the Observational Routinely collected health Data (RECORD) checklist.

The NAR started collecting data on shoulder arthroplasties in 1994. All hospitals in Norway performing SAs report to the register (Fevang et al. 2009). After each operation the surgeon fills in a 1-page paper form, which includes details on the surgical procedure and implants with catalogue numbers. In addition, the form includes information on age, sex, indication for operation, duration of surgery, and intraoperative complications including major bleeding. From 2005 information also includes details on chemical thromboprophylaxis and comorbidity according to the ASA classification. The completeness of reporting of primary SAs in the NAR was 95% for primary operations compared with the Norwegian Patient Registry in 2017–2018 (Furnes et al. 2020).

All patients operated on with SA in the period studied were included regardless of the cause for operation. Rheumatoid arthritis, psoriatic arthritis, ankylosing spondylitis, seronegative arthritis, and systemic lupus erythematosus were grouped together and categorized as inflammatory arthritis. Several diagnoses could be given for each operation, and in cases with more than 1 diagnosis we used the hierarchy developed by the Nordic Arthroplasty Register Association (NARA) (Rasmussen et al. 2016).

The NAR uses the unique personal ID given to each inhabitant of Norway to link the primary shoulder arthroplasty to subsequent revisions and reoperations. Revisions and reoperations are reported equivalent to the primary operation. A revision is defined as the insertion, exchange, or extraction of any of the prosthesis components while a procedure without insertion, exchange, or extraction of components is registered as a reoperation. Multiple reasons for revision can be marked on the form. In cases with more than 1 reason for revision the hierarchy developed by the NARA group was used to determine 1 main reason for revision. Reoperations without the exchange or extraction of components were reported to the register from 2011. In our dataset there were no reported reoperations.

The NAR was linked to the National Population Register and information on death and emigration was available for all patients. Deaths in the first 90 days after surgery were defined as primary outcome, as deaths after this period were considered less likely to be related to the index procedure. Reported intraoperative bleeding complications and revisions during the first year after surgery were also included in the analyses.

All 6,972 primary shoulder arthroplasties reported to NAR in the period 2005–2018 were eligible for inclusion in the study. No patients emigrated during the study period. We excluded 849 operations with missing information in one or more of the variables of interest. Finally 6,123 cases were included in the study.

### Statistics

Pearson’s chi-square test was used for comparison of categorical variables.

Survival time for the 2 subgroups of patients was calculated using Kaplan–Meier estimates. Endpoint was death of any cause within 90 days. Cox regression analyses were used to calculate hazard ratios (HRs) for postoperative deaths and risk of revision between patients receiving thromboprophylaxis and those not receiving prophylaxis, with adjustments for possible confounding of age, sex, ASA score, diagnosis, type of implant (anatomic total, reversed, or hemiarthroplasty), fixation (cemented or uncemented humerus stem), duration of surgery, and year of surgery.

Bilateral cases were treated in the descriptive part as if they were independent, while the adjusted HRs were calculated using robust variance estimates to account for bilateral SAs. Calculation of the robust variance estimates follows the counting process formula of Andersen and Gill (Andersen and Gill 1982, Therneau and Grambsch 2000).

As an alternative to the adjusted Cox regression, we estimated the causal effect of thromboprophylaxis using an instrumental variable (IV) approach. This analysis follows the methods described by MacKenzie et al. (2014) for IVs in a Cox regression model using the statistical package R (R Foundation for Statistical Computing, Vienna, Austria). As instrument, we applied the hospital’s annual propensity for using thrombosis prophylaxis. Hence, the IV approach assumes that the hospital is related to the mortality only through the use of thrombosis prophylaxis, and that the hospital is independent of unobserved covariates. Under these conditions the estimated HR can be interpreted as a causal HR of thrombosis prophylaxis on mortality.

All tests were 2-sided and p-values below 0.05 were considered statistically significant.

Follow-up started on the day of the primary arthroplasty and ended on the date of death or at 90 days for the mortality analyses and at 1 year after surgery for the revision analyses. All analyses were repeated stratifying on age, sex, ASA classification, diagnosis, and arthroplasty type in order to study the potential differences in effect of thromboprophylaxis on outcomes in subgroups of patients.

Analyses were performed using the package IBM SPSS statistics version 24.0 (IBM Corp, Armonk, NY, USA) and the statistical package R Version 4.0.0 (R Foundation, Vienna, Austria).

### Ethics, funding, and potential conflicts of interests

The NAR has permission from the Norwegian Data Inspectorate to collect patient data based on written consent from the patients (ref 24.1.2017: 16/01622-3/CDG). The Norwegian Arthroplasty Register is financed by the Western Norway health authorities. The authors declare no conflict of interest. 

## Results

4,089 cases received thromboprophylaxis and 2,034 did not receive thromboprophylaxis. Low molecular weight heparin (LMWH) was the dominant medication used. 2,778 patients were treated with dalteparin and 1,201 patients with enoxaparin (68% and 29% of the patients receiving thromboprophylaxis respectively).

Patient and procedure characteristics for the 2 groups are shown in Table 1. The patients receiving thromboprophylaxis had statistically significantly higher mean ASA class and longer mean duration of surgery. Patients operated on with a reverse shoulder arthroplasty (RSA) more frequently received thromboprophylaxis compared with patients operated on with stemmed hemiarthroplasty (SHA) and total shoulder arthroplasty (TSA) (p < 0.001).

**Figure 1.**
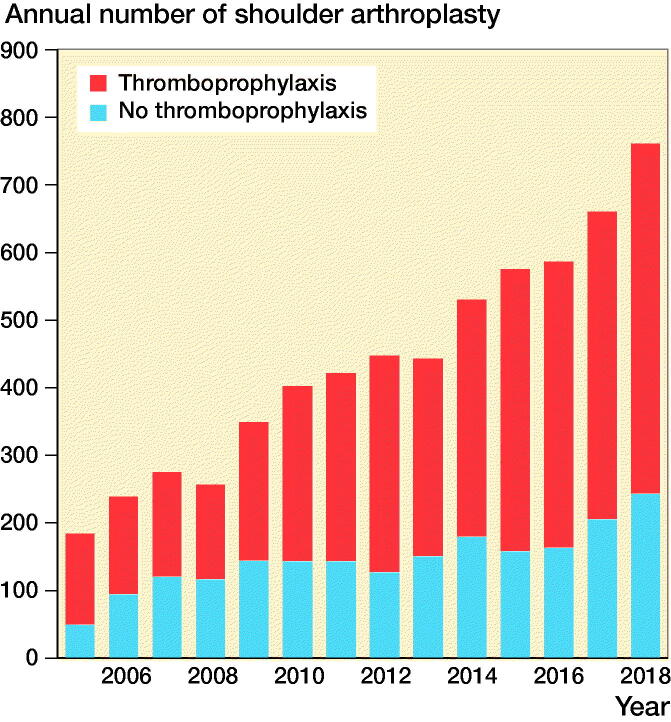
Change in the use of thromboprophylaxis over time, Norwegian Arthroplasty Register 2005–2018.

**Figure 2.**
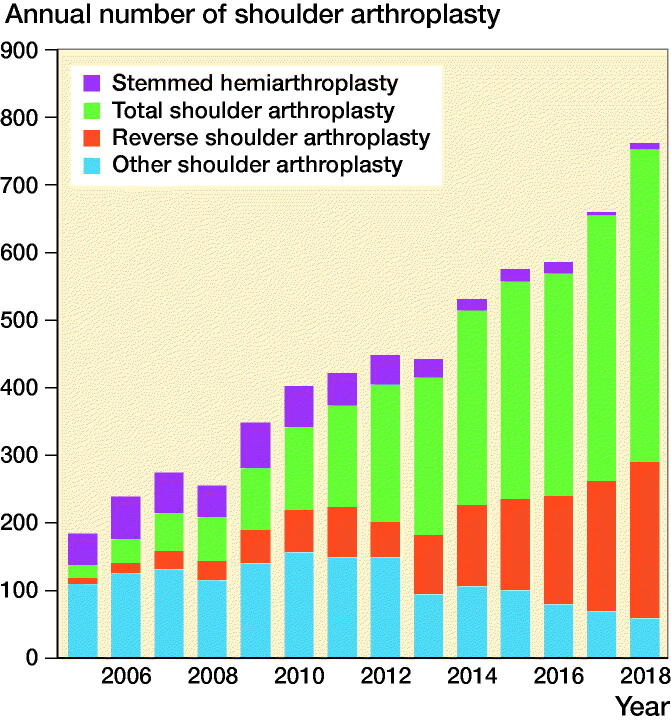
Change in the use of different arthroplasty design over time, Norwegian Arthroplasty Register 2005–2018.

There was an increase in the use of thromboprophylaxis over time in the period studied (Figure 1). The use of hemiarthroplasty dominated in the earlier years of this period, and the use of RSAs and TSAs increased in the later years (Figure 2).

### Risk of death

**Figure 3.**
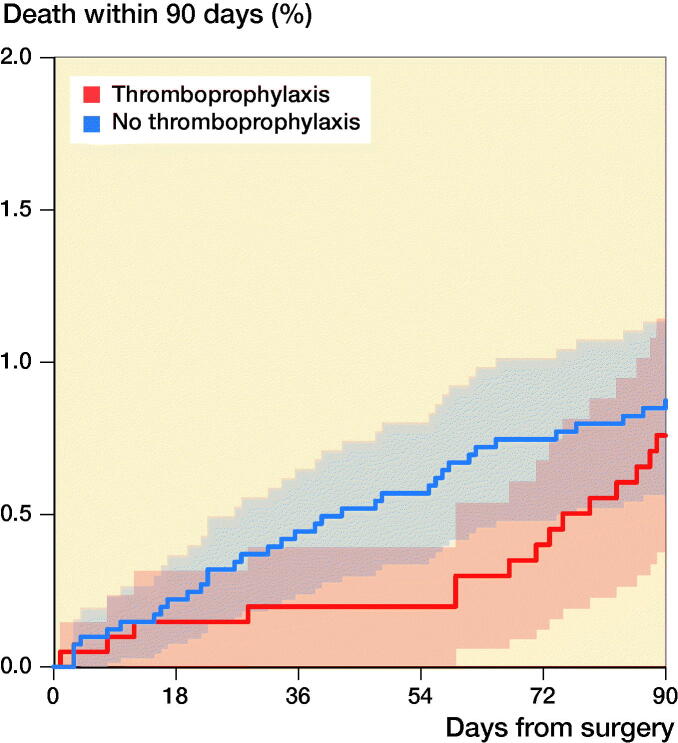
Kaplan–Meier curve showing the death rate up to 90 days after surgery in patients with and without thromboprophylaxis with 95% CI.

We identified 50 deaths within 90 days in the period studied, 35 in the thromboprophylaxis group and 15 in the group with no thromboprophylaxis (Figure 3). Adjusted HR showed no significant difference between the two groups (HR 1.2; CI 0.6–2.2) with the no thromboprophylaxis group as reference. Using the IV approach, we found a non-significant causal effect of thromboprophylaxis on 90-day mortality (HR 1.1; CI 0.6–2.4) (Table 2).

Compared with patients with primary osteoarthritis, patients with acute fractures had a higher 90-day mortality (HR 3.4; CI 1.2–9.5). A similar tendency was found for patients with sequelae after fracture, but the difference was not statistically significant. Patients with rotator cuff arthropathy or inflammatory arthritis did not have increased 90-day mortality compared with patients with primary osteoarthritis (Table 3, see Supplementary data). We found higher 90-day mortality after fracture-related surgery (acute fracture and fracture sequelae) than after non-fracture-related surgery 1.6% (CI 1.0–2.2) vs. 0.3% (CI 0.1–0.5).

Old age (> 75 years), high ASA class (≥ 3), and acute fracture diagnosis statistically significantly increased 90-day mortality. The risk of death was not significantly changed in the different time periods studied (Table 3, see Supplementary data).

### ASA classification and age

Since both increasing ASA class and high age increased mortality, we also performed Cox regression analysis with patients stratified into 3 different risk groups, dependent on both age (≥ 80 based on the Norwegian guidelines for thromboprophylaxis) and ASA classification. This analysis suggested an even stronger correlation between age, ASA class, and the risk of death. We found no statistically significant difference in the distribution of thromboprophylaxis in the different risk groups and use of thromboprophylaxis did not alter the risk of death at 90 days (Table 4, see Supplementary data).

### Revision risk

There were 155 revisions within the first year. Of these, 29 revisions were performed due to deep infections (16 in the thromboprophylaxis group and 13 in the no thromboprophylaxis group). 62 revisions were due to loosening of 1 or more of the components without deep infection recorded. Risks of revision of any cause (HR 0.8; CI 0.6–1.1) and for infection (HR 0.6; CI 0.3–1.2) were similar between the study groups (Table 5, see Supplementary data, Figure 4). No reoperations were recorded.

**Figure 4.**
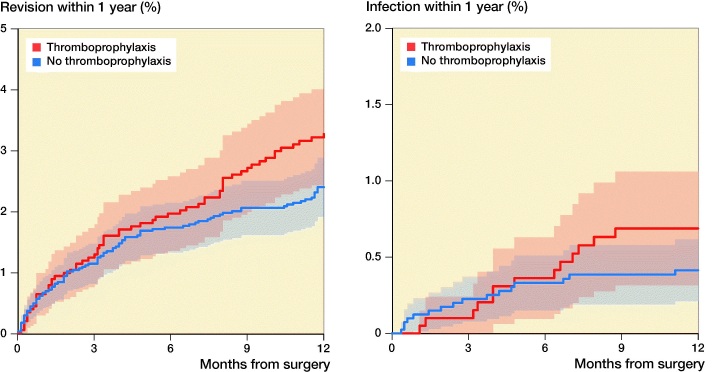
Kaplan–Meier curve showing the revision rate due to all causes (A) and due to infection (B) up to 1 year with 95% CI.

### Intraoperative complications

182 intraoperative complications were registered. Extensive intraoperative bleeding was reported in 17 cases, 12 in the thromboprophylaxis group (0.3%) and 5 in the no thromboprophylaxis group (0.2%). Only 3 of the 12 patients with extensive bleeding in the thromboprophylaxis group had preoperative initiation of the thromboprophylaxis.  

## Discussion

Our main finding was that there was no association between the use of thromboprophylaxis and the risk of death in the postoperative period. As expected, we found that high age, high ASA class, and fracture diagnosis (acute fracture and fracture sequelae) increased the 90-day mortality. Earlier studies on thromboprophylaxis in shoulder arthroplasty surgery include fewer patients, and even though the number of deaths in our study is low the incidence is comparable to earlier studies.

Thromboprophylaxis after shoulder surgery is still a controversial issue: the national guidelines in Norway and other countries are vague. The guidelines in the United Kingdom (National Institute for Health and Clinical Excellence 2018) recommends that the surgeon “Consider VTE prophylaxis for people undergoing upper limb surgery if the person’s total time under general anaesthetic is over 90 minutes or where their operation is likely to make it difficult for them to mobilise.” Based on these recommendations the vast majority of shoulder arthroplasty patients will require thromboprophylaxis. However, VTE events are rare after planned shoulder surgery (0.01–0.5%) (Lyman et al. 2006, Jameson et al. 2011, Navarro et al. 2013).

In the study from Jameson et al. (2011) the 90-day mortality rates after planned shoulder surgery were low (0.03–0.5%), and no change in the mortality rate after the introduction of the 2007 NICE guidelines could be found. Our results with 0.3% 90-day mortality in non-fracture SA surgery support Jameson’s findings.

VTE events are more common in the proximal humerus fracture setting (0.4–1.7%) (Navarro et al. 2013) but compared with other orthopedic procedures the risk is still low (Dahl et al. 2003).

In a large cohort study from Young et al. (2015) proximal humerus fracture, anemia, congestive heart failure, and chronic lung disease were 4 independent predictors for PE after shoulder arthroplasty. As expected, increasing age, fracture diagnosis, and high ASA class correlated with increased mortality in our cohort.

We found increased use of thromboprophylaxis in shoulder arthroplasty surgeries during the studied period. The Norwegian guidelines (Kristiansen et al. 2014) indicating that thromboprophylaxis should be used have probably led to some hospitals changing their use of prophylaxis, but some hospitals were not consistent in their use of prophylaxis. This may be explained by surgeon’s preference or by lack of routines. It could also reflect diversified treatment where patients considered at risk are given thromboprophylaxis. The use does not seem to correlate with the patient’s ASA class, but the ASA class does not fully account for risk factors like previous DVT or other predisposing factors and may therefore not necessarily be a good measure of the actual risk of VTE and mortality. In our study the use of different arthroplasty types changed during the period studied and some of the differences in the use of thromboprophylaxis in different arthroplasties can be explained by the change of indications for the arthroplasty type.

The lack of consensus on the use of prophylaxis in shoulder replacement surgery is reflected by our data, where some hospitals seem to give thromboprophylaxis as a routine and others do not. Some hospitals perform more elective surgery and have more rheumatoid patients while other perform more fracture surgery, and this may also influence the hospital’s routines for thromboprophylaxis. The cost-effectiveness of daily injections of LMWH has to be considered. It is inconvenient for the patient and resource demanding for the healthcare system if patients cannot administer the injections themselves, and there are potential complications. However, we found no difference in intraoperative bleeding complications between the 2 groups and the use of thromboprophylaxis did not seem to affect the risk of revision due to infection. Kwong et al. (2012) found insufficient data in the literature to confirm or refute the hypothesis that postoperative bleeding due to VTE prophylaxis in hip and knee arthroplasty contributes to increased risk for wound infection.

Navarro et al. (2013) observed no difference in 90-day mortality by procedure type (reverse shoulder arthroplasties, total shoulder arthroplasties, or hemiarthroplasties), but a higher mortality in trauma patients compared with elective in his retrospective database review from the Kaiser Permanente registry. In our cohort we found increased risk of mortality in the acute fracture setting, and use of thromboprophylaxis did not alter this risk. Navarro found that only 1 of the 13 deaths observed in his study could be attributed to complications of PE, and this indicates that this is a fragile group of patients with several comorbidities and increased risk of death. In accordance with this we found increased risk of death in the acute fracture group and also higher age in this group.

By dividing patients into risk groups and combining the ASA classification with age, Dale et al. (2020) showed that high-risk patients had nearly 9 times the risk of adjusted perioperative death after primary total hip arthroplasty compared with low-risk patients. In our study the use of thromboprophylaxis did not alter the risk of death within 90 days in any of the risk groups. This does not support the routine use of thromboprophylaxis to prevent death.

The bilateral observations in register studies can be dealt with in different ways (Ranstam et al. 2011). Also, Lie et al. (2004) studied the influence of bilateral hip arthroplasties on survival analyses and concluded that in analyses of arthroplasty survival dependencies should be considered, but ignoring the possible dependencies does not necessarily have an impact on the result. We performed Cox regression analyses with robust variance analyses to account for the bilateral cases and found only small differences (statistically non-significant) between unadjusted and adjusted risk of death. Using an instrument variable analysis approach to estimate the causal effect of thrombosis prophylaxis confirmed the results from the standard analysis.

### Strengths and limitations

This is a nationwide observational cohort study from the Norwegian Arthroplasty Register. The strengths of a register study are the large number of patients and the possibility to study rare events. All hospitals performing shoulder arthroplasties in Norway are reporting to the register and the completeness of reporting primary cases is 95% (Furnes et al. 2020). Information on death and migration was available from Statistics Norway, allowing for nationwide cohort studies with complete follow-up. We do not, however, have access to the cause of death or readmissions to hospital due to VTE or bleeding in these patients. Lie et al. (2002) studied 67,000 hip arthroplasties and early postoperative mortality by linkage to the cause of death registry. They found that vascular causes of death were commonest, with the subcategory thromboembolic complications as the most frequent cause. Even though we do not have access to cause of death in our material we might assume that thromboembolic complications are also a common cause of death in shoulder arthroplasty surgery. This is confirmed in a study from the Danish Shoulder Arthroplasty Registry (Amundsen et al. 2016). They reported the 90-day mortality and the reasons for death between 2006 and 2012. In their study, approximately 30% of deaths were reported with a cardiac or pulmonary cause. In light of the results from Amundsen’s study we can assume that the number of deaths related to thromboembolic events in our study, with only 35 and 15 deaths in the 2 groups, were low and probably insufficient to make any clear recommendations.

The use of thromboprophylaxis as a standard method of treatment varies among hospitals. This might influence the result, as different surgeons may have different results. The instrumental variable analysis accounted for these differences by applying the hospitals’ propensity for using thromboprophylaxis in the model and the results from the standard analysis were confirmed.

An intraoperative bleeding complication was recorded only if the surgeon considered it to be extensive, and the amount of bleeding was not recorded. The completeness of the registration of complications has not been investigated. The findings regarding intraoperative complications must hence be interpreted with caution, and the incidence of such complications is most likely higher than reported. Until 2011, reoperation due to bleeding or hematoma was not reported to the register unless a revision of the prosthesis was also performed. From 2011 all reoperations should be reported to the register, but the completeness of this registration is not known and may be underreported.

### Conclusion

The use of thromboprophylaxis does not seem to reduce the overall low mortality and the use of thromboprophylaxis as a routine in shoulder arthroplasty surgery to prevent thromboembolic complications leading to death can be discussed. We cannot exclude that subgroups of patients with a high risk of VTE, such as earlier VTE events, may benefit from thromboprophylaxis.

## Supplementary Material

Supplemental Material

## Supplementary data

Tables 3–5 are available as supplementary data in the online version of this article, http://dx.doi.org/10.1080/17453674.2021.1906595
