# Supplementary material for: Thromboprophylaxis in primary shoulder arthroplasty does not seem to prevent death: a report from the Norwegian Arthroplasty Register 2005–2018
Source: Acta Orthop. 2021 Apr 6;92(4):401–7. doi: 10.1080/17453674.2021.1906595 (PMC8381958; doi:10.1080/17453674.2021.1906595)
Supplement: Supplemental Material [file IORT_A_1906595_SM2465.pdf]

# Supplementary data

**Table 3. Patient and procedure related unadjusted and adjusted risk of death at 90 days using a Cox regression model and robust variance estimation to account for bilateral cases, reported to the Norwegian Arthroplasty Register 2005–2018**

| | Unadjusted HR (95%CI) | p-value | Adjusted HR (95%CI) | p-value |
|---|---|---|---|---|
| Age | | | | |
| ≤ 64 years | 1 | | 1 | |
| 65–74 years | 0.9 (0.3–2.7) | 0.9 | 0.9 (0.3–3.1) | 0.8 |
| ≥ 75 years | 3.0 (1.2–7.6) | 0.02 | 3.0 (1.1–7.9) | 0.03 |
| Sex | | | | |
| Male | 1 | | 1 | |
| Female | 0.8 (0.4–1.7) | 0.6 | 0.8 (0.4–1.6) | 0.6 |
| ASA class | | | | |
| 1–2 | 1 | | 1 | |
| 3–4 | 5.6 (2.7–12) | < 0.001 | 5.6 (2.5–12) | < 0.01 |
| Diagnosis | | | | |
| Primary osteoarthritis | 1 | | 1 | |
| Acute fracture | 3.4 (1.1–11) | 0.04 | 3.4 (1.2–9.5) | 0.02 |
| Fracture sequelae | 2.8 (0.8–9.6) | 0.1 | 2.8 (1.0–8.3) | 0.06 |
| Rotator cuff arthropathy | 0.8 (0.1–6.6) | 0.8 | 0.9 (0.1–8.0) | 0.9 |
| Inflammatory arthritis | 0.9 (0.9–8.3) | 0.9 | 0.8 (0.1–6.6) | 0.9 |
| Other | 4.0 (0.8–21) | 0.1 | 4.0 (0.8–21) | 0.09 |
| Arthroplasty type | | | | |
| TSA | 1 | | 1 | |
| RSA | 0.4 (0.1–1.6) | 0.2 | 0.4 (0.1–1.2) | 0.1 |
| HSA | 1.7 (0.4–6.8) | 0.4 | 1.7 (0.5–5.8) | 0.4 |
| Other | 0.8 (0.1–7.2) | 0.8 | 0.7 (0.1–7.4) | 0.8 |
| Cemented humeral stem | | | | |
| Yes | 1 | | 1 | |
| No | 0.9 (0.4–1.9) | 0.8 | 0.9 (0.4–1.8) | 0.7 |
| Duration of surgery in min. | | | | |
| ≤ 90 | 1 | | 1 | |
| 91–120 | 1.0 (0.5–1.9) | 1.0 | 1.1 (0.6–2.2) | 0.7 |
| ≥120 | 0.6 (0.3–1.3) | 0.2 | 0.7 (0.3–1.5) | 0.3 |
| Time period | | | | |
| 2005–2010 | 1 | | 1 | |
| 2011–2013 | 0.7 (0.3–1.5) | 0.4 | 0.7 (0.3–1.6) | 0.4 |
| 2014–2016 | 1.0 (0.5–2.2) | 1.0 | 1.0 (0.5–2.1) | 1.0 |
| 2016–2018 | 1.4 (0.6–3.6) | 0.4 | 1.4 (0.6–3.6) | 0.4 |

TSA = total shoulder arthroplasty, RSA = reverse shoulder arthroplasty, SHA = stemmed hemiarthroplasty.

**Table 4. Risk of death at 90 days stratified by risk groups defined by age and ASA class, adjusted for age, sex, ASA class, diagnosis, arthroplasty type, use of cement in humerus, duration of surgery and time period, Cox regression model using robust variance estimation, shoulder arthroplasties reported to the Norwegian Arthroplasty Register 2005–2018**

| | No thromboprophylaxis | | Thromboprophylaxis | | | |
|---|---|---|---|---|---|---|
| Risk group [a] | Deaths at 90 days | At risk at 90 days | Deaths at 90 days | At risk at 90 days | HR [b] (95% CI) | p-value |
| Low | 0 | 1,082 | 4 | 2,098 | – [c] | |
| Intermediate | 6 | 667 | 15 | 1,355 | 1.4 (0.5–3.6) | 0.5 |
| High | 9 | 179 | 16 | 406 | 0.9 (0.4–2.2) | 0.9 |

[a] Risk group defined as Low = age < 80 years and ASA ≤ 2,
Intermediate = age ≥ 80 or ASA ≥ 3, High = age ≥ 80 and ASA ≥ 3.
[b] Cox adjusted hazard ratio (HR) with no thromboprophylaxis as reference
[c] In the no thromboprophylaxis group there were no deaths in the low-risk group and HR is not calculated.

**Table 5. Kaplan–Meier (K–M) estimated risk of revision at 1 year due to all causes and due to infection, shoulder arthroplasties reported to the Norwegian Arthroplasty Register 2005–2018**

| Revision at 1 year | Use of thrombo-prophylaxis | Revisions at 1 year | At risk at 1 year | K–M % revision (95% CI) | Adjusted HR (95% CI) | p-value |
|---|---|---|---|---|---|---|
| All causes | Yes | 63 | 1,694 | 3.3 (2.5–4.1) | 1 (ref.) | |
| | No | 92 | 3,407 | 2.4 (2.0–2.8) | 0.8 (0.6–1.1) | 0.2 |
| Due to infection | Yes | 13 | 1,694 | 0.7 (0.3–1.1) | 1 (ref.) | |
| | No | 16 | 3,407 | 0.4 (0.2–0.6) | 0.6 (0.3–1.2) | 0.2 |

Cox adjusted HR with robust variance estimates adjusted for age, sex, ASA class, diagnosis, arthroplasty type, use of cement in humerus, duration of surgery and time period.
